# Thalamic Aphasia: a Review

**DOI:** 10.1007/s11910-022-01242-2

**Published:** 2022-11-16

**Authors:** Merve Fritsch, Ida Rangus, Christian H. Nolte

**Affiliations:** 1 Department of Psychiatry and Psychotherapy, Charité—Universitätsmedizin Berlin, Campus Mitte, Charitéplatz 1, 10117, Berlin, Germany; 2 Department of Neurology, Charité—Universitätsmedizin Berlin, Berlin, Germany; 3 Center for Stroke Research Berlin, Charité—Universitätsmedizin Berlin, Berlin, Germany

**Keywords:** Thalamic aphasia, Thalamo-cortical language networks, Subcortical aphasia, Lexical-semantic deficits

## Abstract

**Purpose of Review:**

Thalamic aphasia is a rare language disorder resulting from lesions to the thalamus. While most patients exhibit mild symptoms with a predominance of lexical-semantic difficulties, variations in phenotype have been described. Overall, the exact mechanisms of thalamic aphasia await empirical research. The article reviews recent findings regarding phenotypes and possible underlying mechanisms of thalamic aphasia.

**Recent Findings:**

Variations in phenotype of thalamic aphasia may be related to different lesion locations. Overall, the thalamus’ role in language is thought to be due to its involvement in cortico-thalamic language networks with lesioning of certain nuclei resulting in the diachisis of otherwise interconnected areas. Its possible monitoring function in such a network might be due to its different cellular firing modes. However, no specific evidence has been collected to date.

**Summary:**

While recent findings show a more distinct understanding of thalamic aphasia phenotypes and possible underlying mechanisms, further research is needed. Additionally, as standard language testing might oftentimes not pick up on its subtle symptoms, thalamic aphasia might be underdiagnosed.

## Introduction

Thalamic aphasia describes aphasic syndromes stemming from lesions to the thalamus. Aphasia is a clinical syndrome of acquired speech impairments that typically result from lesions to the left hemispheric cortico-subcortical language network [1, 2]. Aphasia is often characterized by the language domains that are primarily affected, and well-established aphasia screening tools use cutoffs in its diagnosis [3]. For example, the “Boston” classification divides aphasia based on deficits in fluency, naming, repetition, comprehension, reading, and writing—with global aphasia describing a loss of all six functional domains [4]. Historically, aphasia was thought to only present after fronto-temporo-parietal cortical lesions and was separated into “Broca’s” and “Wernicke’s” type, referring to a predominant affection of “motor,” or “sensory,” aspects of language [5, 6]. Today, however, it is widely accepted that aphasia may also result from lesions to white matter tracts and subcortical areas [7–12]. Here, aphasia can be due to ischemic or hemorrhagic strokes, as well as tumors, or infections of, e.g., basal ganglia or the thalamus [13–15]. Additionally, language disturbances can occur as a result of stereotactic thalamic and subthalamic deep brain stimulation (DBS) surgery in, e.g., Parkinson’s or Essential Tremor patients, or during thalamotomy [16, 17, 18••, 19, 20]. However, the exact roles of the thalamus and other subcortical structures in language functions are still unclear [7, 18••]. The thalamus is mainly known for its relay function, conducting and modulating afferent signals to the cortex and between different cortical areas—thereby acting as a “gate-keeper” to what we feel and experience [21, 22]. Thus, lesions to the thalamus can result in sensory deficits and neglect, but also in visuo-spatial deficits, pain-syndromes, or even hemiparesis [23]. Additionally, thalamic lesions can lead to a multitude of attentional and cognitive deficits such as amnesia, vigilance impairment, executive dysfunction, apraxia, anosognosia, behavioral and mood alterations, as well as aphasia [24–26]. Similar to its role in sensory processing, the role of the thalamus in language is suggested to be that of a moderator conducting the transfer of lexical information to cortical areas [27]. Thalamic lesions may therefore result in a metabolically or functional decoupling of remote, but interconnected areas that are critical for language function [28]. Such a disconnection, where lesioning of an area, e.g., the thalamus, leads to a dysfunction in another non-lesioned area, for example left cortical structures, or in fact, in a network of brain regions, such as the cortico-thalamic language network, has also been termed diachisis [29].

Here, we review the existing literature on thalamic aphasia. Specifically, we (a) introduce its clinical presentation and phenotypes, (b) describe the involvement of specific, anatomic thalamic subregions, and (c) present its putative underlying mechanisms.

## Clinical Presentation and Phenotypes of Thalamic Aphasia

Thalamic aphasia is less rare than one might expect. With thalamic stroke amounting to 2–4% of all ischemic lesions, and estimation of aphasia after thalamic stroke varying between 12 and 80%, we can expect about 0.25–3.2% of all stroke patients to be affected [23, 30••, 31]. Variation in the estimation of frequency seems to depend largely on clinical language assessments used—with more detailed testing (for example, *Aachener Aphasie Test*, AAT, compared to *National Institute of Health Stroke Scale*, NIHSS) leading to higher detection rates, which, in turn might bias results [25, 30••]. Recovery from thalamic aphasia is often quick, with patients showing little to no symptoms after days or weeks [32–34]. Nevertheless, some case series have also described patients to suffer from persistent aphasic symptoms [35•]. Research suggests that speed and success of aphasia recovery might depend on lesion location and size, although it is unclear whether the same factors are important in thalamic lesions [36].

**Fig. 1:**
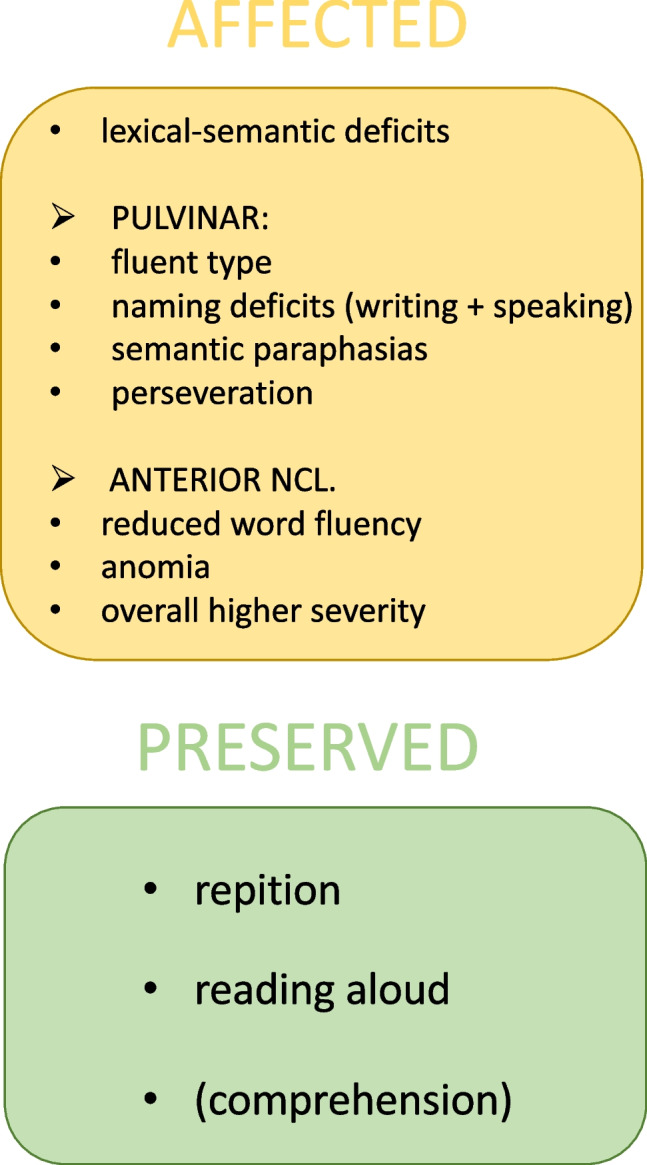
Overview of different language domains predominantly affected or preserved in thalamic aphasia as suggested by the current literature. While lexical-semantic deficits are described after lesions to almost all areas to the thalamus, some symptoms seem to be more common after lesions to specific thalamic nuclei, although more thorough analyses are needed. For example, lesions to the pulvinar are more often associated with fluent aphasia including naming deficits and semantic paraphasias, while lesions to the anterior nuclei can lead to reduced word fluency and show an overall higher severity. However, many phenotypical variations beyond this classification exist

A first description of a thalamic aphasia phenotype came from Crosson in 1984, who reviewed the literature of the time, comprising thalamic aphasia after ischemic or hemorrhagic lesions to be consisting of “[1] word substitutions in spoken language, primarily semantically in nature, [2] jargon in narrative discourse, consisting of words from the patient’s native language, [3] comprehension less impaired than spoken output, and [4] minimally impaired repetition” [37]. Several studies have since evaluated thalamic aphasia using different language assessment tools, and to date, several features of what may characterize it have been discussed. While clinical descriptions differ, and no specific phenotype has been singled out, the literature points towards certain language domains that seem specifically affected, reminiscent of the clinical phenotype initially suggested by Crosson [38]. Overall, aphasia after thalamic lesions has mostly been described as mild, with predominantly lexical-semantic deficits [35•, 39••]. This means that patients exhibit anomia, or naming deficits, in speaking and writing while substituting with semantic paraphasias, i.e., word substitutions with a semantic relationship to the target word, as well as perseverations [25, 30••, 40–43]. In other words, patients make semantically related, but incorrect, word choices and tend to repeat certain words [3]. In most patients that were severely affected, so-called “jargon” language was described, where speech is fluent, but senseless [44]. On the other hand, repetition of words, reading aloud and comprehension, is often intact [24, 27, 30••, 45, 46]. Note, however, that studies using the “Boston” classification have also shown dysfunctional comprehension after ischemic thalamic lesions [38]. Additionally, impaired word fluency is described in some cases, while other studies also reported patients to have intact and fluent spontaneous speech [34, 47]. While these findings appear contradictory at first, it seems likely that certain thalamic areas encode for, or are involved in, different language domains—leading to different clinical presentations in aphasic syndromes, depending on lesion location (Fig. 1) [35•, 39••].

In an early case series, categorizing deficits after thalamic ischemic lesions according to the involved vascular territories, Bogousslavsky et al. described “dysphasia” with reduced verbal output, paraphasias, and impairment of comprehension, albeit preserved repetition, in four patients with left-sided lesions in the tuberothalamic arterial territory, which typically supplies the ventral anterior and ventral lateral nucleus [47]. They additionally described impaired consciousness with subsequent dysphasia and amnestic deficits in patients with paramedian lesions, supplied by the thalamoperforating artery and involving the intralaminar and dorsomedial nuclei. While this stresses the notion of anatomically distinct phenotypes, the latter observation furthermore poses the question whether thalamic aphasia is a byproduct of vigilance impairment rather than a language disturbance in itself. For example, Mohr et al. have described a strong fluctuation in language disturbances in patients following thalamic hemorrhage, with symptoms only occurring in association with vigilance impairment or apathy, which is in line with a more recent hypothesis that thalamic language properties are *mechanistically* similar to its monitoring function of vigilance states [28, 48]. It must be noted that since vascular supply varies greatly, even an ischemic lesion within the same vascular territory might lead to distinct symptoms in different patients [23, 31, 49••]. On the other hand, some studies reported very similar aphasic syndromes despite patients displaying large discrepancies in thalamic lesion location [34]. Critically, however, a relevant amount of the reported data has been collected prior to MRI being routinely available, and neurocognitive assessment was rarely standardized. Thus, the variability in reported syndromes and lesion locations may be partially attributed to varying imaging techniques and quality of language and cognitive assessment.

While there is considerable variability in symptomatology, a basic concept of thalamic aphasia seems to evolve around lexical-semantic difficulties, i.e., finding exact word forms for specific semantic concepts, which is suggested to arise from impaired lexical-semantic retrieval, cf. further explanations below [37]. This notion is additionally supported by stimulation studies in patients undergoing DBS surgery for Essential Tremor or Parkinson’s [16]. Here, specific tasks, which are thought to assess the activation, retrieval, or violation of semantic representations, are associated with thalamic activity [18••, 19, 50–52]. Similarly, studies using functional MRI have reported thalamic activation specifically during object recall or verbal fluency tasks, which are also designed to evaluate lexical-semantic retrieval [1, 53, 54]. Halari et al. additionally saw greater activation during word generation than during repetition, further stressing the thalamus’ possible role in lexical-semantic retrieval and matching the proposed phenotype of thalamic aphasia with preserved repetition [55]. While a strong case can be made for the role in lexical-semantic retrieval, another recent notion has been proposed that primarily higher order language skills, such as in figurative language, synonym, and antonym generation, or in ambiguous sentences are impaired in patients with thalamic lesions [30••, 56]. Ketteler et al. suggested that the thalamus might aid specifically in language processing, such as ambiguity resolution, where more “automated mechanisms,” such as semantic priming, are not precise enough [57, 58]. Similarly, some imaging studies only saw thalamic activation in addition to cortical activation during tasks with higher levels of language difficulty [1, 59, 60]. In summary, current research supports the notion that the thalamus serves specifically during higher-order language functions.

This might have important implications for diagnostic tools for assessment of thalamic aphasia. For instance, patients that display significantly impaired word retrieval do not necessarily meet the cutoff criteria for “aphasia” in standard diagnostics, such as the “standard language test of aphasia” (SLTA) [32, 61]. This underlines the idea that typical routine language assessments might underestimate the subtle impairments in patients with thalamic aphasia [24, 32]. So even when standard diagnostic tools are administered, thalamic aphasia might be underdiagnosed, a concern that could be addressed by more sensitive aphasia examinations, or a stronger focus on separate language domains in the assessment of thalamic aphasia.

## Anatomy of Thalamic Aphasia

As noted above, differences in clinical phenotype of thalamic aphasia may be related to the extent and location of thalamic lesions. While it remains unclear whether specific thalamic nuclei or areas are important for language function “on their own,” or if it is rather their involvement in thalamo-cortical language networks, certain nuclei are thought to have more predominant language properties than others [35•].

### Involved Thalamic Nuclei

With regard to thalamic nuclei, it has been proven difficult to assign distinct thalamic areas to specific language functions, as converging inputs from all neocortical areas to the thalamus, as a whole, exist [62]. It is, however, likely that a nucleus’ function is closely related to the cortical area, with which it is directly connected [63]. Numerous accounts have been made for the thalamic nuclei *most likely* involved in language, and studies have further tried to assign specific aphasia phenotypes to lesioned areas.

Due to their extensive projections to frontal or temporo-parietal cortical areas, the anterior, ventral, and centromedian nuclei, as well as the lateral posterior thalamus and the pulvinar, are thought to be critical for language function [27, 64]. Accordingly, lesions studies have shown aphasia primarily after lesions to the left anterior and inferolateral nuclei, and in lesions to the, overall much more infrequently affected, pulvinar [24, 25, 30••, 40, 42, 65–67]. Additionally, lesions to the right, left, or both paramedian thalamic areas can lead to aphasic as well as complex neurocognitive symptoms [34, 42, 68]. Additional evidence for the importance of the anterior thalamic nuclei in language comes from functional MRI studies showing left and bilateral anterior thalamic activation during a semantic object activation task [69]. Moreover, a systematic pattern of event-related potentials is consistently reported in the vicinity of the ventral intermediate nucleus (VIM) during tasks testing semantic and syntactic rules, which authors have suggested to originate from the anterior or centromedian nuclei [19, 70]. Furthermore, verbal fluency, which has been associated with lexical-semantic retrieval, was reduced in bilateral stimulation of VIM in DBS treatment of Essential Tremor, with the effect being correlated to the frequency of stimulation further pointing to a frequency-modulated informational transfer via the thalamus [16, 17]. It has furthermore been suggested that the anterior thalamic nuclei’s role in the access of lexical-semantic representations is conducted via theta-band activity, which has been shown in electroencephalographic recordings during word retrieval [71–73]. Regarding the ventral anterior thalamus (VA), functional imaging studies provided further insight by showing direct connectivity of the VA to the pre-supplementary motor area (pre-SMA) and Broca’s area [74]. The rostrally located pulvinar holds equally strong projections to temporoparietal cortices and Broca’s area [75]. As resting state functional connectivity of the left pulvinar with left temporo-parietal cortices has been shown to correlate with picture naming, the pulvinar is similarly suggested to be important for lexical-semantical retrieval [76, 77]. It is furthermore hypothesized that the pulvinar may be important in lexical discrimination, as it holds similar properties in visual discrimination; however, no evidence towards this notion has been collected to date [35•, 78].

As mentioned above, when comparing aphasic phenotypes between different thalamic nuclei, slight differences are reported in the literature. While lesions to the pulvinar are described to rather result in a fluent aphasia with semantic paraphasias and naming deficits, lesions to the anterior and ventrolateral nucleus may lead to a non-fluent aphasia type with anomia and more severe overall language deficits [30••, 35•, 41, 67, 76, 77, 79]. Different phenotypes have also been attributed to a difference in involvement of the mentioned nuclei in specific brain networks, an aspect that will be discussed in detail below [39••, 49••].

Again, some limitations need to be mentioned. Most of the aforementioned studies are based on small samples with differing quality of language assessment and imaging techniques, rendering specific differentiations of phenotypes difficult. Additionally, as not all areas of the thalamus are equally likely to be affected by ischemic stroke due to its vascular supply and stimulation studies are mostly reporting on nuclei that are of interest in the pathology and treatment of Parkinson’s and Essential Tremor, a selection bias must be expected [16, 23]. Therefore, existing literature might not give a sufficiently accurate representation of thalamic areas relevant for language processing, with the question, which nuclei are specifically involved to what degree, remaining. Ultimately, evidence suggests that lesion location and size do not predict severity and phenotype of aphasia, both in subcortical and cortical stroke [80].

### Lateralization

Another key question remaining to date is, whether language is lateralized in the thalamus, paralleling its cortical organization, where in right-handed, and most left-handed, subjects language areas are typically located in the left hemisphere [3]. Here, studies of patients with thalamic strokes, or following DBS placement, have reported aphasia to be predominantly associated with lesions, or electrode placement, in left thalamic areas [16–20, 24, 81•]. For instance, in a study with 52 thalamic stroke patients, aphasia, as defined by the utilized aphasia check list (ACL), was associated with lesions in nearly all thalamic regions in the acute-stroke-phase. Intriguingly, however, left anterior lesion location resulted in the most severe deficits, with a predominance of naming difficulties and reduced verbal fluency [30••]. This finding is corroborated by fMRI studies showing predominantly left-sided thalamic activity in language tasks [62]. The notion of such a lateralization of language in the thalamus is challenged by studies showing bilateral [54], or even only right-thalamic activity during active or passive language tasks [55]. Unfortunately, handedness was often not tested, and language impairment was often assessed in patients with left-sided thalamic lesions only, limiting these results [24, 38]. Additionally, selection criteria are not always precisely reported, and such results might partially be due to a selection bias, as more patients with left sided (thalamic as well as cortical) lesions are being detected and hospitalized overall [82].

## Mechanisms of Thalamic Aphasia

So far, most concepts on thalamic aphasia have suggested the role of the thalamus in language to be that of a monitor or conductor in a thalamo-cortical language network, with lesions of the thalamus leading to a disconnection between network structures and thereby to aphasia [45, 49••, 83].

### Studies of Functional and Structural Connectivity and Diachisis

Many of the studies already mentioned in this review have evaluated structural and functional connectivity between thalamic nuclei and cortical areas in association with language functions, thereby stressing the idea of thalamo-cortical networks in language [1, 53, 54, 59, 74]. Furthermore, studies specifically showing anatomic, metabolic, or functional disruption within networks after thalamic lesions bring even further insight. This so-called diachisis, originally describing an anatomical disconnection, is today understood as a multi-factorial deterioration of a non-lesioned area due to lesioning in another, remote location based on changes in their network communication [29, 84].

One hypotheses suggests that subcortical lesioning leads to temporary cortical hypoperfusion and/or hypometabolism and thereby to aphasia [85, 86]. This could explain the short-lived nature of thalamic aphasia, as duration of this disruption may be temporary [85]. Using positron emission tomography (PET) and tractography in six patients, Nishio et al. were able to show that left anterior thalamic infarctions lead to a disruption of metabolic and fiber connections between the anterior thalamus and fronto-temporal cortical areas, which was associated with lexical-semantic deficits [43, 65]. Stenset et al. described a similar hypometabolism with reduced left fronto-parieto-temporal cortical glucose uptake in PET as well as reduced fractional anisotropy (FA) after a left anterior thalamic lesion corresponding to disturbances in naming as well as in semantic and working memory which persisted over at least 6 months in one patient [87]. Baron et al. reported a correlation of aphasia, though not specified, and other neurocognitive impairments with ipsilateral, cortical hypometabolism, as seen in PET, after thalamic lesions with improvement in symptoms paralleling changes in glucose uptake [88]. In a longitudinal study using PET in a small cohort of seven patients with subcortical strokes and aphasia (predominantly anomia), De Boissezon showed a reduction of regional cerebral blood flow (rCBF) in the left fronto-temporal cortex following stroke [89]. While not all patients had exclusive lesions in the thalamus, the authors suggested the observed recovery in clinical and imaging parameters being associated with a functional diachisis phenomenon. It has further been hypothesized that (transient) aphasia after thalamic lesions might be due to transient cortical hypoperfusion associated with cerebral artery stenosis [90–92]. While Hillis et al. were able to associate specific language impairments with cortical hypoperfusion patterns in perfusion MRI in patients with non-specific subcortical lesions, Sebastian et al. only reported regional, cortical hypoperfusion in one of five patients with thalamic aphasia (showing fluent speech with naming errors) [90, 92]. Furthermore, in a single photon emission computed tomography (SPECT) and near infrared spectroscopy (NIRS) study, Obayashi et al. (2022) reported that thalamic lesions led to hypoperfusion of language-related left cortical areas such as the supplementary motor area (SMA) and the frontal cortex that would normally be connected by the frontal aslant track (FAT). Interestingly, as low SMA activity correlated with poor word retrieval, high SMA activity was observed in recovery of the same patients during follow-up [32]. While this study is limited by its small sample size, the results suggest a critical role of the SMA frontal cortex loop via FAT in aphasia after thalamic lesions. Notably, also cortical lesions have been reported to relate to changes in the thalamo-cortical language network. In post-stroke aphasic patients with left cortical lesions, increased intra-thalamic functional connectivity in the right thalamus was associated with more severe symptoms, specifically naming deficits [93]. Furthermore, Keser et al. were able to show that even non-lesioned left thalamo-cortical pathways degenerated in patients with aphasia after left-cortical strokes which, again, correlated with severity of naming deficits [94•]. Similarly, Guo et al. described aphasic patients to have an increase in functional connectivity of the right thalamus in a thalamo-cortical language network, possibly correlated to semantic processing [95]. This stresses the previously mentioned evidence on a thalamo-cortical language network being involved in naming and lexical-semantic properties and overall suggests that thalamo-cortical connections play a critical role in the pathology and recovery of aphasia.

### Mechanistic Models

While a network of cortical and thalamic areas in language and other cognitive tasks seems evident as such, the underlying mechanisms have not been fully understood and remain a challenge for ongoing research. Many considerations regarding possible models have proposed similar ideas of a modulating or orchestrating effect on cortical activity. Since a specific aspect of thalamic aphasia seems to be lexical-semantic disturbances, a focus often lies on explaining the role of the thalamus in lexical-semantic retrieval. In general models of language pathology, lexical-semantic errors are thought to result from damage to the access, or storage, of preformed lexical-semantic representations, which can follow ischemic or other lesions to fronto-temporo-parietal cortical areas [96]. Even early concepts of thalamic aphasia have considered similar aspects. Ojemann et al., Wallesch and Papagno, or Fristen et al. proposed that thalamic activity would not just trigger unspecific arousal, but might activate specific, relevant cortical circuits needed for language functions and support in the selection of one of multiple lexical alternatives that were previously generated in the cortex [12, 45, 97, 98].

The idea that the thalamus helps in “choosing the right word” has also been discussed recently. DBS stimulation of VIM in Essential Tremor patients can lead to a shift towards more frequent words, similar to observations made in dementia, which is considered a marker for a “lexical simplification” of language—possibly due to dysfunctional lexical retrieval [18••]. As missing words are then substituted by semantically similar, but more frequent words, this may lead to the evolution of semantic paraphasias often observed in thalamic aphasia [49••]. Correspondingly, Nadeau and Crosson (1997) suggested that the thalamus “selectively engages” cortical areas critical for operations during lexical access [27]. As part of two proposed cortico-subcortical loops (“frontal lobe – inferior thalamic peduncle – reticular nucleus – centromedian nucleus” and “cortex – pre-SMA – Brodmann’s area 32 – dorsal caudate nucleus – ventral anterior thalamus”), thalamic nuclei may specifically access neuronal networks, leading to the release of cortically generated language segments—an execution that becomes dysfunctional in thalamic lesions [27, 49••]. The authors refined this idea in the “Response Release Semantic Feedback” Model, suggesting that the thalamus, and other basal ganglia, scale, and sequence language options before speech, is then actually produced [64]. A possible computational and mechanistic explanation for such a scaling and selection processes in language is provided in the “Lexical Selection Model” by Norris et al. They describe lexical, auditory recognition as a Bayesian inferential process in which word recognition is based on a comparative evaluation of multiple, lexical hypotheses—a process that could be based on a computational information transfer between cortex and subcortical regions [99, 100]. Relatedly, Crosson proposed a feedforward and feedback mechanism of “word selection” between cortico-cortical and cortico-thalamic networks, in which higher-order thalamic relays maintain a stable representation of known lexical information, which are perpetually compared with possible semantic solutions for a given object, proposed by the cortex. Mismatches of the object with proposed lexical forms may then lead to error signals and to suppression of possible incorrect choices. In a step-wise process of iterations of this kind, the most likely word choice would be refined and emerge [101]. The thalamus could herby aid in enhancing resolution especially in lexical ambiguity in more difficult language tasks by suppressing incorrect choices for words [57]. Taking a similar computational approach to language, the “Declarative/Procedural” model proposes an additional separation of language functions into memorized lexical formations and the underlying, grammatical rules needed to form larger words or sentences [39••, 102]. Similar to the memory system, both aspects are thought to be encoded by different neural circuits: the temporal cortex holding representation for the declarative system, and a frontal cortex-basal ganglia loop encoding for procedural memory [39••, 102]. It remains unclear, however, whether the thalamus acts merely as part of a basal-ganglia loop in procedural memory aspects of language (i.e., grammar) or whether it is also involved in declarative-lexical processes [39••]. Crosson recently proposed that the pulvinar might be involved in declarative processes while the dorsal-medial nucleus could be involved in grammatical operations. He further suggests that the ventral anterior and lateral nuclei could be involved in lexical search by suppressing competing word alternatives [39••]. This could again also explain why damage to different thalamic nuclei might lead to different forms of aphasia (as mentioned above). In summary, different hypothetical and computational models suggest possible involvements of the thalamus in language. However, an exact mode of action has yet to be discovered. Therefore, further research is needed to show the exact role of the thalamus in language processing, and ultimately, in aphasia.

### The Role of Different Cell Types

A possible underlying mechanism, conducting the aforementioned selective recruitment of cortical areas in a language network, might be the specific neuronal organization and firing modes within the thalamus. Here, thalamic nuclei can be categorized into first-order relay nuclei, receiving input mainly from subcortical sources, and transferring sensory information to the cortex, and higher-order relay nuclei that conduct informational transfer from one cortical area to another [63, 103]. First-order relay cells are known to exhibit different firing modes, which in turn have an important impact on the sleep–wake-cycle [104]. While rhythmic, oscillatory activity in the delta band range stabilizes sleep and shields the organism from incoming sensory information, a tonic spiking mode is adopted in awake states to promote precise and linear information transfer to the cortex [105]. Higher-order thalamic relays make up most of the thalamus’ volume and are thought critical in transthalamic informational transfer to the cortex in cognitive tasks and memory [103, 106]. Such informational transfer, possibly via different firing modes, may be similarly driving thalamic recruitment of cortical areas, such as pars triangularis of Broca’s area, for language [49••, 107]. This notion is supported by studies suggesting an importance of specifically higher-order thalamic relay activity in other cognitive tasks such a learning, decision-making, and goal-directed behavior [108, 109]. While similar processes in language functions seem plausible, no direct evidence has been collected to date as data on different cell types or firing modes in the specific setting of thalamic aphasia are scarce and further research is needed.

## Conclusions

Thalamic aphasia is relatively rare, with 0.25 to 3% of ischemic stroke patients being affected. While phenotype and severity can vary greatly, most patients typically exhibit mild symptoms, with a predominance of lexical-semantic deficits, such as anomia and paraphasias, while comprehension and repetition are mostly spared. Prognosis is overall good with most patients recovering quickly. Anatomically, lateralization of language function to the left thalamus has been widely suggested, especially in lesion studies, but some indication of additional right-sided and bilateral thalamic activity during language tasks prevails. While aphasia has been seen after lesions in almost all thalamic regions, the anterior nuclei and the pulvinar have been specifically singled out as important in language function and might have specific importance for certain language domains. Most mechanistic models on thalamic aphasia suggest these thalamic areas to be part of cortico-thalamic language networks with lesions to the thalamus leading, i.e., to a diachisis of the interconnected areas and thereby to deficits in lexical-semantic retrieval. The thalamus’ role in activating specific cortical areas needed for language could be explained due to its different cellular firing modes, with so-called higher-order thalamic nuclei, possibly driving this recruitment of areas involved in the language network. Of clinical importance, thalamic aphasia is probably underdiagnosed, as symptoms are often mild and might not be picked up by standard language testing. To submit the affected patients to the best possible care, more precise and differentiated language assessment is needed in clinical routine and in future studies examining thalamic aphasia.
